# DHX9 phosphorylation at S321 by ATM regulates DHX9 retention at DNA double-strand break sites and interaction with BRCA1

**DOI:** 10.1016/j.jbc.2025.110526

**Published:** 2025-07-25

**Authors:** Saaya Matsuya, Yuina Tsuchiya, Yudai Hiwatashi, Yurina Abe, Ryotaro Nishi

**Affiliations:** 1 Graduate School of Bionics, Tokyo University of Technology, Hachioji, Tokyo, Japan; 2 School of Bioscience and Biotechnology, Tokyo University of Technology, Hachioji, Tokyo, 192-0982, Japan

**Keywords:** homologous recombination, DNA–RNA helicase, DHX9, phosphorylation, DNA double-strand break

## Abstract

To preserve genome stability, the repair of DNA double-strand breaks (DSBs) that can be caused by exposure to ionizing radiation and certain anticancer drugs is of paramount importance. Recently, it became evident that various DNA–RNA helicases play a pivotal role in homologous recombination (HR) repair and non-homologous end joining, which are the two principal DSB repair machineries in mammalian cells. In a previous study, we reported that DHX9, which belongs to the DExH-box helicase family, is involved in HR repair. However, the regulatory mechanisms governing the function of DHX9 remains elusive. The present study has demonstrated that upon etoposide treatment, DHX9 was phosphorylated at S321 in a manner dependent on ataxia telangiectasia mutated (ATM), a protein kinase. In addition, cell cycle synchronization and fractionation analysis of cell extracts revealed that only chromatin-bound DHX9 was phosphorylated by ATM in the S phase, where HR repair functions. Furthermore, by live-cell imaging with unphosphorylated-mutant and phospho-mimic DHX9, we revealed that the S321 phosphorylation of DHX9 was required for the retention of DHX9 at DSB sites but not for the initial recruitment of DHX9 to DSB sites. The DSB repair efficiencies were found to be reduced in both cell lines expressing either the unphosphorylated mutant or the phospho-mimic DHX9. Consistent with this, phospho-mimic DHX9 showed reduced interaction with BRCA1. In conclusion, our findings indicate that the DSB-induced ATM-dependent phosphorylation of DHX9 at S321, which should be dynamically regulated, is crucial for efficiency of the DSB repair.

The genomic DNA is constantly threatened by various endogenous and exogenous factors, such as DNA replication collapse, reactive oxygen species, ultraviolet light, and ionizing radiation (1, 2). Among DNA lesions that are generated by these factors, DNA double-strand break (DSB) is one of the most deleterious types of DNA lesion. To avoid unfavorable effects caused by DSBs, DSBs are repaired by a series of DSB repair mechanisms that have different characteristics. In mammalian cells, DSBs are predominantly repaired by non-homologous end joining (NHEJ) or homologous recombination (HR) repair, especially synthesis-dependent strand annealing that is a subpathway of HR in somatic cells (3, 4, 5, 6, 7, 8). While NHEJ relatively quickly ligates two DSB ends with minor processing that can cause deletion or insertion of nucleotides at a DSB site, HR is high-fidelity repair mechanism since DNA sequence of undamaged sister chromatid is copied during HR. Consistent with these characteristics of NHEJ and HR, DSBs generated in the coding region of mammalian genome tends to be repaired by HR, indicating a pivotal role of HR for preserving genomic information (9, 10). Mechanistically, HR is initiated with degradation of a DNA strand in 5′ to 3′ direction on the both sides of DSB, which is known as DNA end resection (11). DNA end resection is carried out by orchestrated reaction of MRE11–RAD50–NBS1 complex, CtIP, Exonuclease1, Bloom syndrome protein, DNA2, and a series of accessory factors. Generated 3′ overhanged single-strand DNA is coated with RPA (RPA1–RPA2–RPA3 complex), which is replaced by RAD51 subsequently. RAD51-bound single-strand DNA invades into a sister chromatid, and DNA synthesis is carried out. Since the long stretch of single-strand DNA is formed in HR, HR and NHEJ are mutually exclusive. Therefore, the choice of DSB repair mechanism is crucial for preserving mammalian genome integrity. The mechanism regulating the choice of DSB repair mechanisms has been extensively studied in a large number of laboratories, and it is well known that the accumulation of either 53BP1 or BRCA1 at the site of damage promotes NHEJ or HR, respectively (12). In an attempt to elucidate preferential choice of HR in the coding region, we and Hiom’s group previously revealed that DHX9 (also known as RHA and NDHII), a DNA–RNA helicase, promotes HR by facilitating loading of BRCA1 to the sites of DSB (13, 14). Our study suggested that nuclear speckle that is a membraneless nuclear body and resides next to transcriptionally active loci play important roles in the favorable choice of HR (14). However, how the DHX9 function is regulated during HR remained unexplored.

DSB repair and accompanying cellular responses to DSB such as cell cycle checkpoint are tightly regulated by various protein post-translational modifications (PTMs) including phosphorylation that was one of the first reported PTMs regulating DSB responses (15, 16, 17, 18). Three kinases function at the early stages of DSB responses, namely ataxia telangiectasia mutated (ATM), DNA-dependent protein kinase catalytic subunit (DNA-PKcs), and ataxia telangiectasia and Rad3-related (ATR), which belong to phosphatidylinositol 3-kinase–related kinase family and phosphorylate serine (S) or threonine (T) followed by glutamine (S/TQ) (19). These kinases regulate DSB repair, DSB repair pathway choice, and DSB responses by phosphorylating a wide variety of substrates by altering protein–protein interaction and subcellular localization. DHX9 participates in transcription, DNA replication, translation, and antiviral response in addition to HR (20) and receives some PTMs including ubiquitylation and phosphorylation (21, 22, 23, 24); however, whether DHX9 is under control of phosphorylation in HR is not investigated. In this study, we revealed that DHX9 interacts with ATM and is phosphorylated on S321 in ATM-dependent manner upon DSB induction. Furthermore, during S phase, only DHX9 in chromatin fraction is phosphorylated, whereas in G1 phase, predominantly soluble DHX9 was phosphorylated. Importantly, DHX9 phosphorylation was required for stable binding of DHX9 to the site of damage. Furthermore, phospho-mimic DHX9 showed weakened interaction with BRCA1, suggesting that DHX9 phosphorylation should be dynamically regulated during HR. Taken together, we identified novel DHX9 regulatory mechanism by phosphorylation in HR.

## Results

### DHX9 was phosphorylated upon induction of DSB

**Figure 1:**
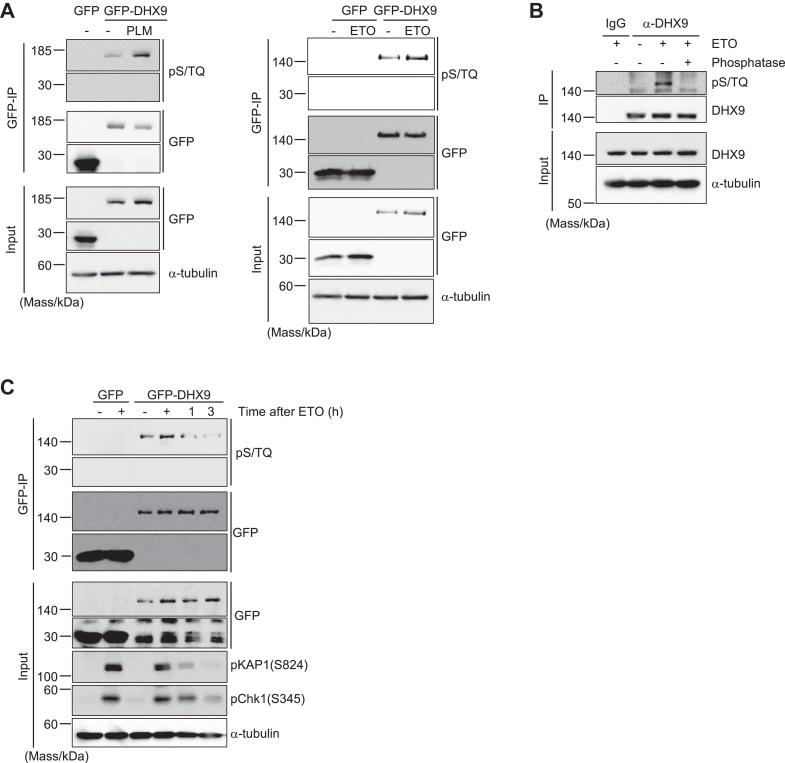
**DHX9 was phosphorylated in response to induction of DSB.***A*, U2OS cells transiently expressing either GFP or GFP-DHX9 were mock treated (−) or treated with 500 μM phleomycin (PLM, *left*) for 2 h or 50 μM etoposide (ETO, *right*) for 1 h. Input soluble fraction and immunoprecipitated fractions by anti-GFP antibody–conjugated beads (GFP-IP) were analyzed by immunoblotting with the indicated antibodies. *B*, soluble fractions of U2OS cells treated with 50 μM ETO for 1 h or mock treated were subjected to immunoprecipitation with either anti-DHX9 antibody (α-DHX9) or control IgG. Where indicated, immunoprecipitated sample was incubated with phosphatase. Input fraction and immunoprecipitated fraction were analyzed by immunoblotting with the indicated antibodies. *C*, U2OS cells stably expressing either GFP or GFP-DHX9 were mock treated (−) or treated with 50 μM ETO for 1 h (+). Following wash out of ETO, cells were further cultured for 1 or 3 h. Soluble fractions were subjected to immunoprecipitation with anti-GFP antibody and then analyzed by immunoblotting with the indicated antibodies. DSB, DNA double-strand break; GFP-DHX9, GFP-tagged DHX9.

DHX9 was previously shown to be post-translationally modified under various circumstances (21, 22, 23, 24); however, whether DHX9 receives PTMs in the context of DSB repair was not investigated. Since phosphorylations by phosphatidylinositol 3-kinase–related kinases such as ATM intensely regulates DSB repair, DHX9 phosphorylation was investigated after treating cells with either phleomycin (radio-mimetic drug) or etoposide (topoisomerase II inhibitor) that induce DSBs. Immunoblotting analysis with anti-phospho S/TQ antibody of immunoprecipitated GFP-tagged DHX9 (GFP-DHX9) showed that DHX9 was phosphorylated at the S/TQ motif after these treatments (Fig. 1*A*). Importantly, endogenous DHX9 that was immunoprecipitated with the anti-DHX9 antibody was also phosphorylated in response to etoposide treatment, which was diminished by treating immunoprecipitated GFP-DHX9 with phosphatase (Fig. 1*B*). To speculate the role of DHX9 phosphorylation in DSB repair, we also tested how the phosphorylation of DHX9 changes after DSB induction (Fig. 1*C*). The etoposide-induced DHX9 phosphorylation reached a peak immediately after etoposide treatment and was gradually reduced after removal of etoposide, which was similar to the phosphorylation pattern of KAP1 (S824) and Chk1 (S345), which are substrates of ATM and ATR, respectively. These results suggest that DSB-induced DHX9 phosphorylation at the S/TQ motif was a relatively early event in DSB repair and gradually diminished accompanied with the completion of DSB repair.

### DHX9 was phosphorylated at S321 depending on ATM

**Figure 2:**
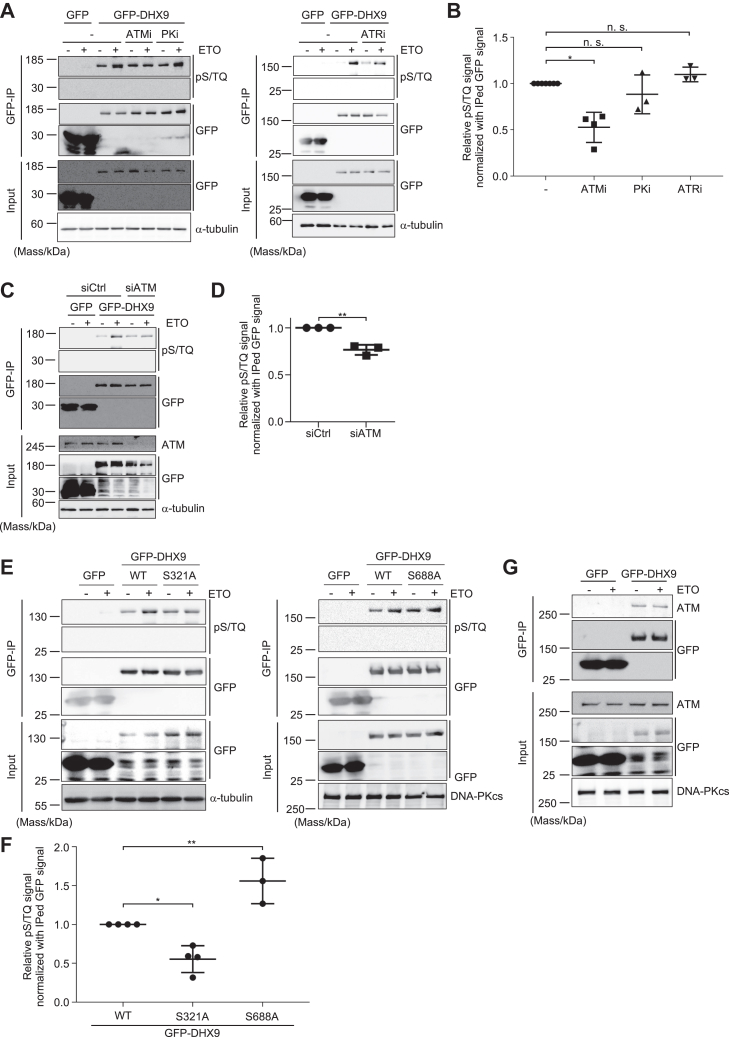
**DHX9 was phosphorylated at S321 by ATM.***A*, U2OS stably expressing either GFP or GFP-DHX9 was incubated with KU55933 (ATMi, 10 μM, 1 h), NU7441 (PKi, 3 μM, 1 h), or VE-821 (ATRi, 1 μM, 1 h) prior to etoposide treatment (50 μM, 1 h). Cells not treated with etoposide (−) were negative controls. Soluble fractions were subjected to immunoprecipitation with anti-GFP antibody and then analyzed by immunoblotting with the indicated antibodies. *B*, quantification analyses of etoposide-treated samples in (*A*). The intensity of phosphorylation signal of DHX9 (WT) in GFP-IP fraction, which was normalized with immunoprecipitated GFP signal intensity, was set to 1.0. The relative intensity of phosphorylation signal of each sample, which was normalized with immunoprecipitated GFP signal intensity as well, was plotted (mean ± SD). ∗*p* < 0.05. ns, not significant. Significance was tested with Dunn’s test. *C*, cells expressing either GFP or GFP-DHX9 were transfected with the indicated siRNAs and then were mock treated (−) or treated with etoposide (50 μM, 1 h). Soluble fractions were subjected to immunoprecipitation with anti-GFP antibody and then analyzed by immunoblotting with the indicated antibodies. *D*, quantification analyses of etoposide-treated samples in (*C*). The intensity of phosphorylation signal of siCtrl in GFP-IP fraction, which was normalized with immunoprecipitated GFP signal intensity, was set to 1.0. The relative intensity of phosphorylation signal of siATM sample, which was normalized with immunoprecipitated GFP signal intensity as well, was plotted (mean ± SD). ∗∗*p* < 0.01. Significance was tested with Student’s *t* test. *E*, cells stably expressing GFP, GFP-DHX9 (S321A), or GFP-DHX9 (S688A) were mock treated (−) or treated with etoposide (50 μM, 1 h). Soluble fractions were subjected to immunoprecipitation with anti-GFP antibody and then analyzed by immunoblotting with the indicated antibodies. *F*, quantification analyses of etoposide-treated samples in (*E*). The intensity of phosphorylation signal of DHX9 (WT) in GFP-IP fraction, which was normalized with immunoprecipitated GFP signal intensity, was set to 1.0. The relative intensity of phosphorylation signal of each sample, which was normalized with immunoprecipitated GFP signal intensity as well, was plotted (mean ± SD). ∗*p* < 0.05, ∗∗*p* < 0.01. Significance was tested with Holm–Sidak’s test. *G*, cells stably expressing either GFP or GFP-DHX9 were mock treated (−) or treated with etoposide (50 μM, 1 h). Soluble fractions were subjected to immunoprecipitation with anti-GFP antibody and then analyzed by immunoblotting with the indicated antibodies. ATM, ataxia telangiectasia mutated; ATR, ataxia telangiectasia and Rad3-related; GFP-DHX9, GFP-tagged DHX9; PK, protein kinase.

We then sought to identify the kinase responsible for DSB-induced DHX9 phosphorylation by incubating cells either with the inhibitor of ATM (KU55933), DNA-PKcs (NU7441), or ATR (VE-821) prior to DSB induction by etoposide. While ATM inhibition significantly reduced etoposide-induced DHX9 phosphorylation, inhibition of DNA-PKcs or ATR had little effect (Fig. 2, *A* and *B*). Consistently, knocking down ATM with siRNA resulted in reduced etoposide-induced DHX9 phosphorylation (Fig. 2, *C* and *D*). Subsequently, we tried to identify at which residues DHX9 gets phosphorylated. For this purpose, a DHX9 hetero knockout cell line (DHX9 hKO) was established by CRISPR–Cas9 technique (a detailed characterization of the cell line is described in the article submitted elsewhere). Using the DHX9 hKO cell line as a parental cell line, stable cell lines expressing GFP-DHX9 that was either WT or mutants in which S321 and/or S688 was replaced by A (S321A, S688A, and S321AS688A [SSAA], respectively) were generated (Fig. S1). Since S321 and S688 residues consisting of SQ motifs were reported as potential phosphorylation sites by several mass spectrometry analyses (25), we decided to focus on these two residues among 11 serine or threonine residues consisting of S/TQ motifs in DHX9. Surprisingly, etoposide-induced DHX9 phosphorylation was increased with DHX9 (S688A) compared with that of DHX9 (WT), whereas DSB-induced phosphorylation was significantly reduced with DHX9 (S321A) mutant (Fig. 2, *E* and *F*), indicating that ATM phosphorylates S321 of DHX9. Finally, if ATM phosphorylates DHX9, an interaction between these two proteins can be detected. Indeed, GFP-DHX9 interacted with endogenous ATM regardless DNA damage (Fig. 2*G*). These results suggested that DHX9 is directly phosphorylated at S321 upon DSB induction by ATM. We also found that DHX9 phosphorylation could be detected without DNA damage even in the presence of ATM inhibitor and after knocking down ATM (Fig. 2, *A* and *C*). Furthermore, phosphorylation signal was still detected with DHX9 mutant (S321A) even in the absence of DNA damage (Fig. 2*E*). However, immunoblotting analysis with the anti-phospho S/TQ antibody did not indicate phosphorylation of endogenous DHX9. Since DHX9 is a multifunctional protein and high expression of DHX9 was suggested to affect cell proliferation (26), exogenously expressed GFP-DHX9 may be phosphorylated during cell proliferation processes, not during DSB repair.

### Chromatin-bound DHX9 was preferentially phosphorylated in S phase

**Figure 3:**
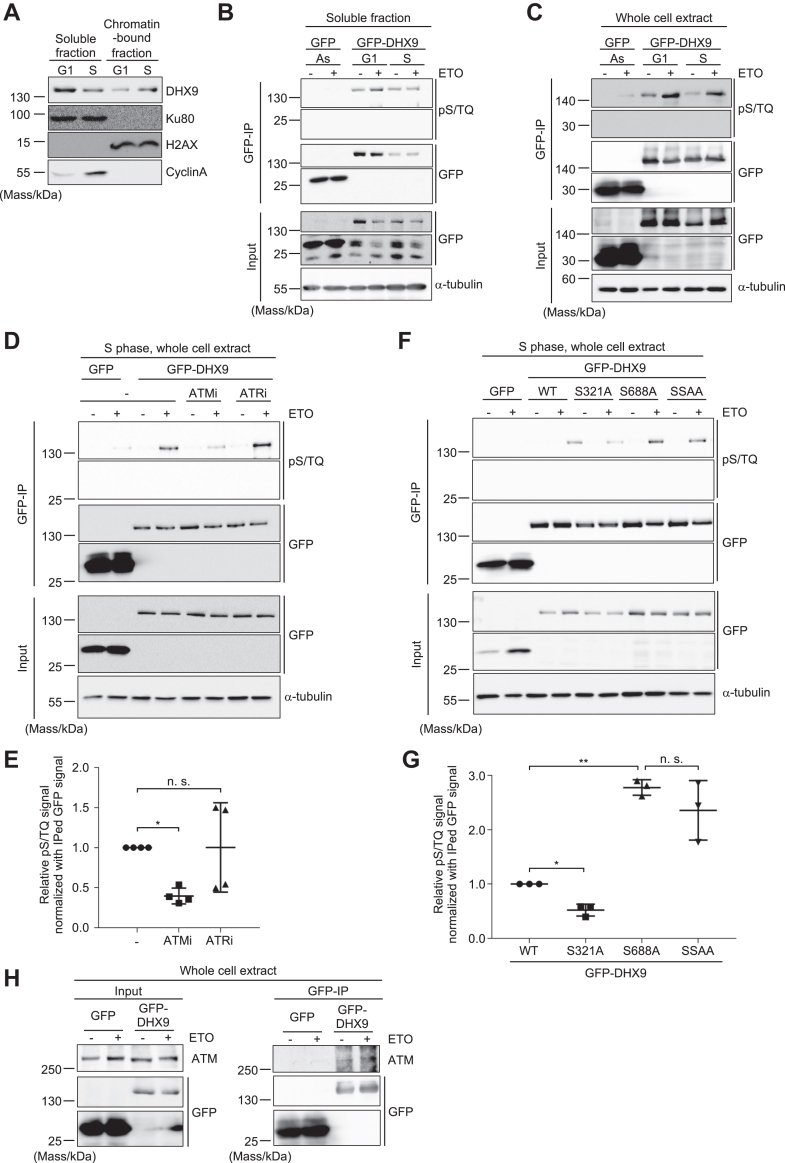
**Chromatin-bound DHX9 was preferentially phosphorylated in S phase.***A*, following synchronization of U2OS cells at the G1 phase or S phase, soluble and chromatin-bound fractions were prepared. Expression of DHX9 was examined by immunoblotting with the anti-DHX9 antibody. Cyclin A detection was used as a control for cell cycle phase. Ku80 and H2AX were loading control for soluble and chromatin-bound fraction, respectively. *B* and *C*, U2OS cells stably expressing GFP-DHX9 were synchronized at the G1 phase or the S phase and then mock treated (−) or treated with etoposide (50 μM, 1 h). U2OS cells stably expressing GFP were subjected to same treatment without cell cycle synchronization (asynchronous [AS]). Soluble fractions (*B*) or whole cell extract (*C*) were subjected to immunoprecipitation with anti-GFP antibody and then analyzed by immunoblotting with the indicated antibodies. *D*, U2OS cells stably expressing either GFP or GFP-DHX9 were synchronized at the S phase and then treated with KU55933 (ATMi, 10 μM, 1 h) or VE-821 (ATRi, 1 μM, 1 h) prior to etoposide treatment (50 μM, 1 h) or mock treatment (−). Whole cell extracts were subjected to immunoprecipitation with anti-GFP antibody. Input fraction and immunoprecipitated fraction were examined by immunoblotting with the indicated antibodies. *E*, quantification analyses of etoposide-treated samples in (*D*). The intensity of phosphorylation signal of DHX9 without inhibitor in GFP-IP fraction, which was normalized with immunoprecipitated GFP signal intensity, was set to 1.0. The relative intensity of phosphorylation signal of each sample, which was normalized with immunoprecipitated GFP signal intensity as well, was plotted (mean ± SD). ∗*p* < 0.05. ns, not significant. Significance was tested with Dunn’s test. *F*, DHX9 hKO cells stably expressing GFP or GFP-DHX9 (WT: WT, S321A, S688A, or S321AS688A: SSAA) were synchronized at the S phase and then treated with etoposide (50 μM, 1 h) or mock treated (−). Whole cell extracts were subjected to immunoprecipitation with anti-GFP antibody. Input fraction and immunoprecipitated fraction were examined by immunoblotting with the indicated antibodies. Mock treatment (−) was also prepared. *G*, quantification analyses of etoposide-treated samples in (*F*). The intensity of phosphorylation signal of DHX9 (WT) in GFP-IP fraction, which was normalized with immunoprecipitated GFP signal intensity, was set to 1.0. The relative intensity of phosphorylation signal of each sample, which was normalized with immunoprecipitated GFP signal intensity as well, was plotted (mean ± SD). ∗*p* < 0.05, ∗∗*p* < 0.01. ns, not significant. Significance was tested with Holm–Sidak’s test. *H*, U2OS cells stably expressing either GFP or GFP-DHX9 were mock treated (−) or treated with etoposide (50 μM, 1 h). Whole cell extracts were subjected to immunoprecipitation with anti-GFP antibody and then analyzed by immunoblotting with the indicated antibodies. ATM, ataxia telangiectasia mutated; ATR, ataxia telangiectasia and Rad3-related; GFP-DHX9, GFP-tagged DHX9.

Although DHX9 has been demonstrated to promote HR that specifically functions in S and G2 phases of the cell cycle (13, 14), whether DHX9 chromatin binding is regulated in the cell cycle–dependent manner, and whether the status of DHX9 chromatin binding affects its phosphorylation, remains unanswered. First, we investigated the status of DHX9 chromatin binding by synchronizing U2OS cells in either G1 phase or S phase. While DHX9 can be detected in both soluble and chromatin-bound fractions in both the G1 and S phases, the chromatin-bound fraction of DXH9 was increased in the S phase, whereas the soluble fraction was reduced, in comparison to the G1 phase (Fig. 3*A* and Fig. S2*A*). Furthermore, the expression of DHX9 protein in relation to the cell cycle was examined using whole cell extract (Fig. S2*B*), indicating that the total protein level was not dramatically altered between the G1 and S phases. Subsequently, etoposide-induced DHX9 phosphorylation was investigated with either soluble fraction (Fig. 3*B*) or whole cell extract (Fig. 3*C*) in each cell cycle phase. Although etoposide-induced DHX9 phosphorylation was detected with both methods in the G1 phase, it was observed only when whole cell extract was used in the S phase (Fig. 3, *B* and *C*). These results indicate that DSB-induced phosphorylation of DHX9 can occur in both the G1 and S phases, but that during S phase, DHX9 phosphorylation occurs specifically on chromatin-bound DHX9. These also indicated that aforementioned DSB-induced DHX9 phosphorylation (Figs. 1 and 2) predominantly reflected DHX9 phosphorylation events occurred in the G1 phase. Consequently, the kinase responsible for DHX9 phosphorylation and the phosphorylation site in the S phase were investigated by solubilizing chromatin-bound DHX9. The inhibition of ATM resulted in an attenuated etoposide-induced DHX9 phosphorylation, whereas ATR inhibition had a minimal effect (Fig. 3, *D* and *E*). The DHX9 (S321A) mutant exhibited significantly reduced level of phosphorylation compared with the DHX9 (WT) (Fig. 3, *F* and *G*). It is worth to note that DHX9 (S688A) and DHX9 (S321AS688A: SSAA) mutants resulted in increased phosphorylation upon etoposide treatment (Fig. 3, *F* and *G*). In addition, the interaction between DHX9 and ATM was also identified in cell extracts prepared with a buffer containing benzonase (Fig. 3*H*), suggesting that the interaction between DHX9 and ATM was not mediated by nucleic acids. These data suggested that DHX9 was phosphorylated on S321 by ATM throughout the cell cycle, but that DHX9 in chromatin-bound or soluble fraction was targeted in the S or G1 phase, respectively. Finally, a comparison of amino acid sequences of *Homo sapiens* DHX9 and its homologs of *Mus musculus*, *Danio rerio*, *Xenopus laevis*, and *Drosophila melanogaster* revealed that the SQ motif containing S321 was well conserved through vertebrates, suggesting the importance of the phosphorylation at S321 (Fig. S3).

### DHX9 phosphorylation was required for stable binding of DHX9 to DSB site

**Figure 4:**
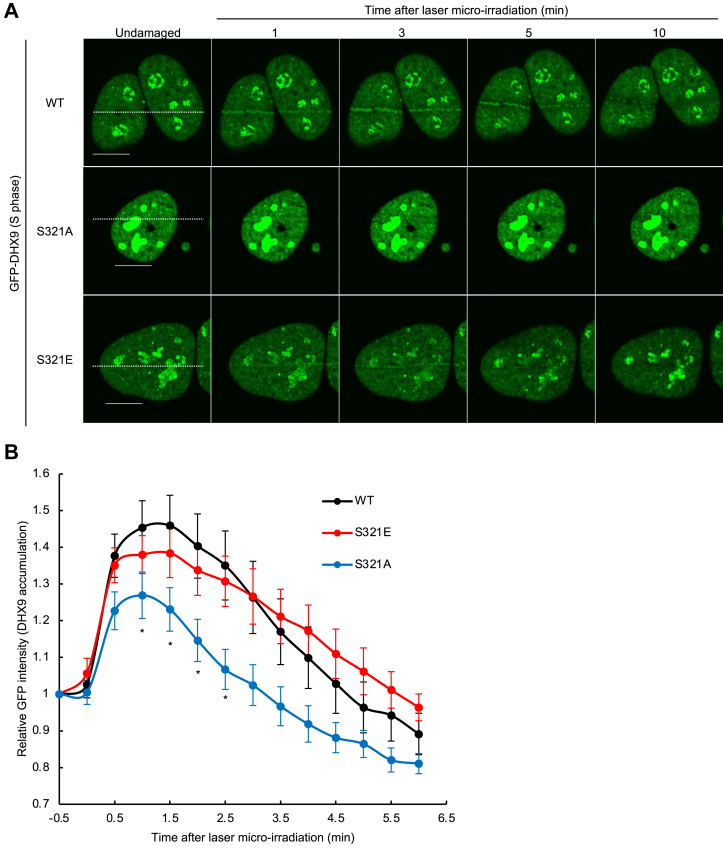
**DHX9 phosphorylation was required for maintaining DHX9 at DSB sites.***A*, U2OS cells transiently expressing GFP-DHX9 (WT, S321A, or S321E) were synchronized at the S phase and subjected to live-cell imaging with laser microirradiation. *Dotted lines* indicate where the cells were laser microirradiated. Scale bars represent 10 μm. *B*, the GFP signal intensity at laser microirradiated area was plotted as a function of time (mean ± SEM, WT: n = 13, S321A: n = 13, and S321E: n = 14). The GFP signal intensity at laser microirradiated area was normalized with the GFP signal intensity of undamaged area. ∗*p* < 0.05. Significance was tested with Student’s *t* test. DSB, DNA double-strand break; GFP-DHX9, GFP-tagged DHX9.

The majority of DSB repair factors are known to be recruited to the site of DSB, which can be detected as the recruitment to laser-induced DSB sites. Given that we recently discovered that DHX9 also can be recruited to 405 nm laser-induced DSB sites (the article was submitted elsewhere), we investigated how phosphorylation of DHX9 affects DHX9 dynamics was investigated using live-cell imaging. GFP-DHX9 (WT) was recruited to laser-induced DSB sites within 2 min of laser irradiation, with the accumulation mostly resolved by 5 min (Fig. 4*A*). This was consistent with the proposed involvement in early DSB repair events (Fig. 1*C*). However, the accumulation of GFP-DHX9 (S321A) to DSB sites was significantly reduced in comparison to the WT, and moreover, this unphosphorylated mutant was even excluded from DSB sites (Fig. 4, *A* and *B*). In contrast, the recruitment of GFP-DHX9 (S321E) to DSB sites was comparable to that of GFP-DHX9 (WT) (Fig. 4, *A* and *B*). These results indicated that phosphorylation of DHX9 on S321 is required for the maintenance of DHX9 at DSB sites but not for its recruitment.

### Dynamic regulation of DHX9 phosphorylation was required for efficient DNA-end resection

**Figure 5:**
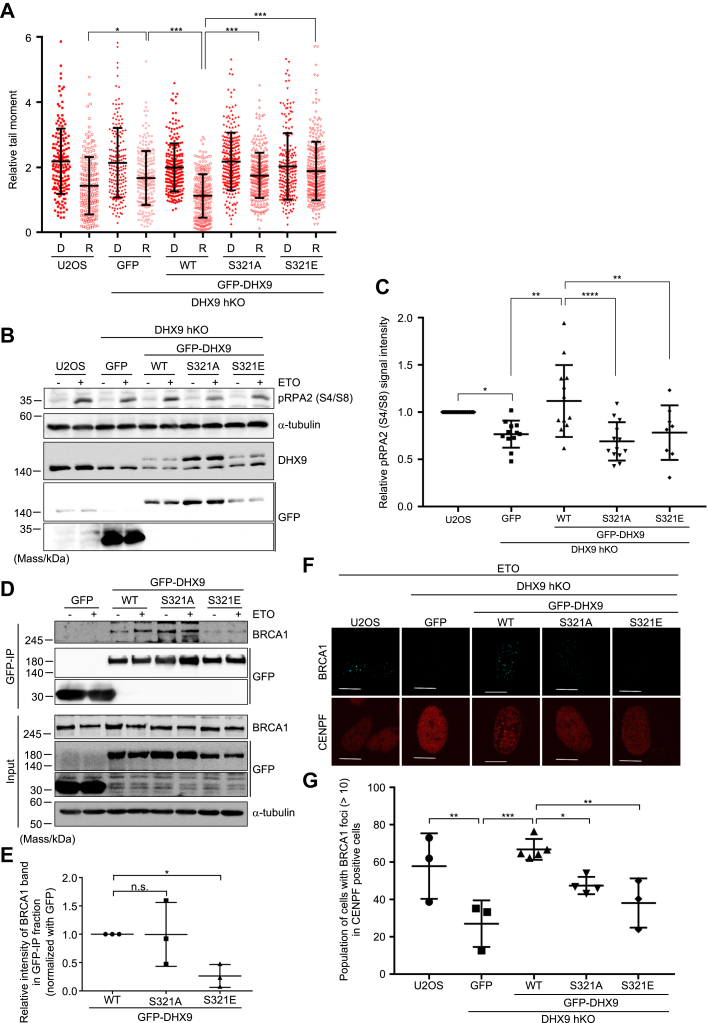
**Dynamic regulation of DHX9 phosphorylation was required for efficient DNA-end resection.***A*, the indicated cells were subjected to neutral comet assay. Relative tail moments that were normalized with the mean of tail moments of undamaged sample were plotted (means ± SD). The tail moments of immediately after phleomycin treatment (Damaged [D]) and of 2 h after removal of phleomycin (Repaired [R]) were shown as closed and opened symbols, respectively. ∗*p* < 0.05, ∗∗∗*p* < 0.0001. Significance was tested with Tukey’s test. *B*, indicated cells were treated with etoposide (50 μM, 1 h) or mock treated (−). Soluble fractions were subjected to immunoblotting analysis with the indicated antibodies. *C*, the signal intensity with anti-pRPA2 (S4/S8) antibody of U2OS cells, which were normalized with the signal intensity of α-tubulin, was set to 1.0. The relative signal intensities with anti-pRPA2 (S4/S8) antibody of each sample, which were also normalized with the signal intensity of α-tubulin, were plotted (mean ± SD). ∗*p* < 0.05, ∗∗*p* < 0.005, and ∗∗∗∗*p* < 0.0001. Significance was tested with Sidak’s test. *D*, DHX9 hKO cells stably expressing either GFP or GFP-DHX9 (WT, S321A, or S321E) were mock treated (−) or treated with etoposide (50 μM, 1 h). Soluble fractions were subjected to immunoprecipitation with anti-GFP antibody and then analyzed by immunoblotting with the indicated antibodies. *E,* quantification analyses of etoposide-treated samples in (*D*). The signal intensity with anti-BRCA1 antibody in GFP-DHX9 (WT)–expressing sample in GFP-IP fraction, which were normalized with the signal intensity of anti-GFP antibody, was set to 1.0. The relative intensity with anti-BRCA1 antibody in each sample, which was normalized with immunoprecipitated GFP signal intensity as well, was plotted (mean ± SD). ∗*p* < 0.05. ns, not significant. Significance was tested with Dunnett’s test. *F*, the indicated cells that were treated with etoposide (20 μM, 1 h) or mock treated were processed for immunofluorescent staining with the indicated antibodies. Immunofluorescent staining with anti-CENPF antibody was used as a marker of cells in G2 phase. Scale bar represents 10 μm. The images of mock-treated cells are presented in Figure S5. *G*, quantification analyses of (*F*). Population of cells with more than 10 BRCA1 foci in CENPF-positive cells was plotted (mean ± SD). ∗*p* < 0.05, ∗∗*p* < 0.01, and ∗∗∗*p* < 0.001. Significance was tested with Holm–Sidak’s test. GFP-DHX9, GFP-tagged DHX9.

Furthermore, the impact of DHX9 phosphorylation on DSB repair was examined by the neutral comet assay (Fig. 5*A* and Figs. S1 and S4). A significant defect in DSB repair was observed in DHX9 hKO cells expressing GFP, which was rescued by complementing GFP-DHX9 (WT), indicating that DHX9 was required for efficient DSB repair, consistent with our previous results (the article was submitted elsewhere). In contrast, cells expressing GFP-DHX9 (S321A) exhibited a markedly delayed DSB repair, suggesting that DHX9 phosphorylation is required for optimal DSB repair efficiency. It was unexpected that cells expressing GFP-DHX9 (S321E) also exhibited defective DSB repair, suggesting that DHX9 phosphorylation should also be appropriately removed during DSB repair. Consistent with the results of DSB repair assay, phosphorylation on S4 and S8 (pRPA2 S4/S8), which was generated during DNA-end resection, was decreased in both cells expressing DHX9 (S321A) or DHX9 (S321E) (Fig. 5, *B* and *C*). To further investigate the significance of DHX9 phosphorylation turnover, we examined its interaction with BRCA1 (Fig. 5, *D* and *E*). DHX9 (WT) and the nonphosphorylatable mutant DHX9 (S321A) showed similar levels of interaction with BRCA1, whereas the phosphomimetic mutant DHX9 (S321E) exhibited reduced binding. In addition, BRCA1 foci formation was attenuated in cells expressing either DHX9 (S321A) or DHX9 (S321E) compared with those expressing WT DHX9 (Fig. 5, *F* and *G* and Fig. S5). On the other hand, the interaction with USP42 was not noticeably affected by the phosphorylation of DHX9 (Fig. S6). In conclusion, these data suggested that DHX9 phosphorylation at S321 was required for stable binding of DHX9 at DSB sites and then should be removed to interact with BRCA1 most likely to facilitate DNA-end resection (Fig. S7).

## Discussion

We and others previously had suggested that DHX9 plays a significant role in promoting HR (13, 14). However, the regulatory mechanisms that govern DHX9 function had not been investigated. In this study, the molecular mechanism of DHX9 phosphorylation and its importance in DSB repair were indicated by a series of evidence. The phosphorylation of DHX9 at S321, which is well conserved throughout vertebrates, was dependent on ATM and occurred in response to etoposide-induced DSB. The physical interaction between DHX9 and ATM suggested that DHX9 could be directly phosphorylated by ATM. Importantly, only chromatin-bound DHX9 was subject to phosphorylation by ATM during the S phase. Furthermore, DHX9 was rapidly recruited to the site of damage (within 2 min after DSB induction), and DHX9 phosphorylation at S321 was necessary for maintaining DHX9 at DSB sites. Consistently, the efficiency of DSB repair and the HR-related signal (pRPA2 [S4/S8]) was reduced in cells that expressed the S321A mutant. The phospho-mimic mutant of DHX9 (S321E) was recruited to and maintained at DSB sites in a similar manner with WT. Surprisingly, the defect of DSB repair and pRPA2 (S4/S8) signal of DHX9 hKO cells were not rescued by expressing the S321E mutants, whereas the WT rescued the phenotype. These results suggest that the phosphorylation of DHX9 that occurs immediately following DSB induction is required for the efficient retention of DHX9 at DSB sites. However, it is also crucial to remove the phosphorylation at an optimal time point. Consistent with this, the interaction between BRCA1 and DHX9 and BRCA1 foci formation were attenuated in cells expressing DHX9 (S321E), suggesting that DHX9 phosphorylation at S321 should be removed to efficiently interact with BRCA1. Altogether, our results indicate that the dynamic regulation of DHX9 phosphorylation, which occurs immediately after DSB induction, is critical for efficient DSB repair.

There are several potential mechanisms through which phosphorylation-dependent retention of DHX9 may facilitate DSB repair. S321 of DHX9 resides in a region between the double-strand RNA-binding domain II and the helicase domain (Fig. S7). The recently solved crystal structure of DHX9 indicated that a region containing S321 did not form a stable structure, suggesting that phosphorylation at S321 may provide specific binding with nucleic acids or DHX9 interactors and promote its retention at DSB site (27). Our results indicated that phosphorylation of DHX9 at S321 was required for retaining DHX9 at DSB sites but negatively affected on the interaction with BRCA1. Therefore, DHX9 may change its function, that is, RNA–DNA helicase and binding platform for BRCA1, by the phosphorylation status. It would be intriguing to examine the structure of DHX9 with nucleic acids or BRCA1. Furthermore, given that a ubiquitin E3 ligase, RNF168, interacts with the N-terminal region of DHX9 (1-404 amino acids) and promotes DHX9 recruitment to R-loops through ubiquitylation (21), it is plausible that S321 phosphorylation may also promote R-loop binding of DHX9 by mediating the interaction between DHX9 and RNF168. In addition to its role in protein–protein interactions, the nucleic acid binding of DHX9 may also be affected by S321 phosphorylation. DHX9 binds various nucleic acid structures, including double-strand DNA, single-strand DNA, double-strand RNA, single-stand RNA, and DNA–RNA hybrid, which can all be present at DSB sites (28, 29, 30, 31). Therefore, S321 phosphorylation may alter the preference nature of the binding of DHX9 to nucleic acids. We also found that S688 of DHX9, which resides within the helicase domain, is important for repressing or regulating DHX9 phosphorylation, suggesting this residue is important for maintaining the proper structure of DHX9 (Fig. S7).

We also have found that chromatin-bound DHX9 was exclusively phosphorylated in the S phase (Fig. 3). This suggests that there will be prerequisites for phosphorylation, which could be S phase–specific PTMs or interacting partners (proteins and RNAs). Furthermore, our data indicated that DSB-induced DHX9 should be removed appropriately (Fig. 5, *B*–*G*), suggesting the involvement of phosphatases in the regulation of DHX9. In order to fully understand the regulation of DHX9, aforementioned unresolved questions will be of interest.

Recently, Liu *et al.* (24)reported that S321 of DHX9 was phosphorylated by ATR in response to genotoxic stress. In their study, topoisomerase I (TOP1) inhibitor, camptothecin, which generates a TOP1 cleavage complex, was employed as the primary agent to induce DNA damage. Although we did not find the effect of ATR inhibition on DHX9 phosphorylation (Figs. 2, *A* and *B*, 3, *D* and *E*), this discrepancy could be attributed to the reagent used to induce DSB. Specifically, while etoposide generates two-ended DSB, camptothecin generates one-ended DSB, which occurs as a result of collision between the TOP1 cleavage complex and the DNA replication machinery. Following the induction of DSB, ATM is activated prior to ATR activation, which requires RPA-bound single-strand DNA that generates *via* DNA end resection (32). In addition, our findings indicate that S321 phosphorylation promotes DHX9 retention at DSB sites immediately after DSB induction (Fig. 4, *A* and *B*). These may suggest that S321 can be phosphorylated both by ATM and ATR in a manner dependent on the structure of DSB ends.

DHX9 is overexpressed in a number of cancers, including small cell lung cancer, colorectal cancer, and hepatocellular carcinoma (33, 34, 35, 36, 37, 38), suggesting that DHX9 may represent a novel target for cancer therapy. However, DHX9 is involved in various cellular functions, including DSB repair, transcription, splicing, DNA replication, and the antivirus response. It will therefore be challenging to target DHX9 directly, and it is crucial to reveal the functional separation, with regard to PTMs.

## Experimental procedures

### Cell lines and cell culture

All cell lines were cultured at 37 °C in a humidified 5% CO_2_ atmosphere. All cells were cultured with Dulbecco’s modified Eagle’s medium (DMEM; Nacalai Tesque) containing 10% fetal bovine serum (FBS; Sigma–Aldrich, Biosera), 100  U/ml penicillin (Nacalai Tesque), 100 μg/ml streptomycin (Nacalai Tesque), and 584 μg/ml l-glutamine. DHX9 hKO cell line was established by transfecting both a vector coding guide RNA and CAS9 (pCAS-Guide; Origene), and a vector containing homology arm and puromycin-resistant gene (pDonor-D09; GeneCopoeia) into U2OS cell. The details of guide RNA sequence and homology arm region were described elsewhere (the article was submitted). Stable cell lines expressing GFP-DHX9 were established by transfecting U2OS or DHX9 hKO with the pEGFP-C1 (Takara Bio USA) vector coding DHX9 followed by selection with 1 mg/ml Geneticine (Nacalai Tesque) for 2 weeks. Where indicated, cells were incubated with the inhibitor of ATM (KU55933; Sigma–Aldrich), DNA-PKcs (NU7441; Sigma–Aldrich), or ATR (VE-821; Selleck Biotechnology) for 1 h prior to etoposide treatment. All cell lines were tested for mycoplasma.

### Transfection of plasmids and siRNAs

Cells were transfected with the plasmid with 1 mg/ml of Polyethylenimine “Max” (PEI-MAX; molecular weight of 40,000; Polysciences) as described later. PEI-MAX (two volumes of used plasmid) was diluted in OPTI-MEM (Thermo Fisher Scientific) and then incubated for 5 min at room temperature. The PEI-MAX containing media were mixed with OPTI-MEM supplemented with the plasmid and further incubated for 20 min at room temperature. Finally, the mixture was added to the cells cultured with DMEM supplemented only with 10% FBS. Cells in 35 or 100 mm dish were transfected with 2 or 12 μg of plasmid, respectively. The siRNAs were synthesized by Eurofins Genomics, and sequences targeting Luciferase or ATM were 5′-CGUACGCGGAAUACUUCGA-3′ and 5′-UGGUGCUAUUUACGGAGCU-3′, respectively. These siRNAs (40 nM at final concentration) were transfected with HiperFect (Qiagen) according to the manufacturer’s instructions.

### Cell extract preparation and immunoprecipitation

For preparing fractionated cell extract, the cells were washed twice with ice-cold PBS (Nacalai Tesque) and incubated with an appropriate volume of CSK buffer (10 mM Pipes [pH 6.8], 3 mM MgCl_2_, 1 mM ethylene glycol Bis(2-aminoethyl ether)-*N*,*N*,*N*′,*N*′-tetraacetic acid, 0.1% Triton X-100, and 300 mM sucrose) containing 300 mM NaCl, 1x Protease Inhibitor (PI) cocktail EDTA-free (Nacalai Tesque), 10 mM NaF (Nacalai Tesque), 20 mM *N*-ethylmaleimide (NEM; Nacalai Tesque), and 0.25 mM PMSF (Sigma–Aldrich) for 1 h on ice with occasional mixing. Soluble fractions were collected by centrifugation at 20,000*g* for 10 min at 4 °C. Residual chromatin-bound fractions were washed twice, and then resuspended with the identical buffer, followed by sonication (UD-100, 40% output, 30 s; TOMY). For whole cell extract preparation, cells were washed twice and collected with an appropriate volume of ice-cold PBS, followed by centrifugation at 10,000*g*, for 1 min. Cells were incubated with 200 μl of IP lysis buffer (20 mM Tris–HCl [pH 7.5], 2 mM MgCl_2_, 0.5% Nonidet P-40 [Nacalai Tesque]) containing 40 mM NaCl, 1x PI, 10 mM NaF, 20 mM NEM, 0.25 mM PMSF, and 50 U/ml Benzonase Nuclease (purity >90%; Merck) for 5 min at room temperature. After addition of 800 μl of IP lysis buffer containing 365 mM NaCl, 1x PI, 10 mM NaF, 20 mM NEM, and 0.25 mM PMSF, lysate was incubated at 4 °C for 1 h. Whole cell extract was obtained collecting supernatant after centrifugation at 20,000*g* for 10 min at 4 °C. Where indicated, cells were incubated with 50 μM etoposide (Tokyo Chemical Industry) for 1 h. The protein concentrations of cell extracts were determined with Protein Assay CBB Solution (Nacalai Tesque) with bovine serum albumin (BSA) as a standard (Takara Bio). The antibodies used for immunoblotting in this research are summarized in Table S1. Soluble fraction or whole cell extract was subjected to immunoprecipitation with an anti-GFP antibody coupled with magnetic beads (GFP-Trap_MA; ChromoTek) by rotating overnight at 4 °C. The beads were washed six times with the buffer used for cell extract preparation, and bound proteins were eluted by boiling at 95 °C for 10 min with 1x Laemmli buffer (62.5 mM Tris–HCl [pH 6.8], 2% sodium dodecyl sulfate, 10% glycerol, 0.02% bromophenol blue, and 6.25% β-mercaptoethanol). For immunoprecipitation of endogenous DHX9, appropriate amount of Protein G Sepharose 4 Fast Flow (Sigma–Aldrich) was incubated with 0.1% BSA/PBS at 4 °C for 1 h and then washed twice with PBS. Washed beads were suspended with the buffer used for whole cell extract preparation. Whole cell extract (1900 μg as protein) was incubated with 3.8 μg of the anti-DHX9 antibody or normal mouse immunoglobulin G (Santa Cruz Biotechnology) overnight. After the addition of Protein G Sepharose 4 Fast Flow, whole cell extract was incubated at 4°C at least for 4 h. The beads were washed six times with the buffer used for whole cell extract preparation, and the bound proteins were eluted by boiling at 95 °C for 10 min with 1x Laemmli buffer. Images were taken with LuminoGraph I (ATTO) and quantified with ImageJ software (National Institutes of Health).

### Cell cycle synchronization and analysis

Cell cycle synchronization was carried out by double-thymidine block method. Cells were incubated with 2 mM thymidine (Nacalai Tesque) for 16 h. Thymidine was removed by washing cells with PBS twice, and then cells were cultured for 10 h without thymidine. Cells were synchronized at G1/S boundary by incubating with 2 mM thymidine for 16 h. For S phase synchronization, cells were cultured for 4 h after the removal of thymidine cells. For G1 phase synchronization, cells were cultured in the presence of 1 ng/ml nocodazole (Sigma–Aldrich) for 16 h after removal of thymidine and then released for an additional 6 h. Cells at each cell cycle phase were collected with appropriate volume of PBS and spin downed at 500*g*, for 5 min. Cells suspended with 200 μl of PBS were fixed with 5 ml of ice-cold 70% ethanol and kept at −30 °C for overnight. After washing with PBS twice, cells were incubated with a solution containing 50 μg/ml propidium iodide (Nacalai Tesque), 0.1 mg/ml RNase A (Takara Bio), and 0.05% Triton X-100 for 30 min at 37 °C. Cell cycle profile was analyzed by flow cytometer (Sony; SH800S).

### Laser microirradiation and live-cell imaging

Cells were seeded into 35 mm glass bottom dish (Matsunami; D11530H) and on the next day transfected with the plasmid coding GFP-DHX9 (WT, S321A, or S321E). Cells were then subjected to S phase synchronization as described previously. On the day of imaging, cells were incubated with 10 μg/ml Hoechst 33342 (Nacalai Tesque) for 15 min, and then the culture medium was replaced with Phenol Red–free DMEM (Nacalai Tesque) supplemented with 10% FBS, 100  U/ml penicillin, 100 μg/ml streptomycin, and 584 μg/ml l-glutamine. Cells were then subjected to laser microirradiation with confocal microscope FV3000 (Olympus) equipped with heat stage (TOKAI HIT). Cells were irradiated with 405 nm laser, and images were taken every minute. Quantification of GFP signal was carried out with ImageJ software.

### Neutral comet assay

The cells were incubated with 40 μg/ml phleomycin (Invivogen) for 2 h or mock treated. Following phleomycin treatment, cells were washed twice with PBS and cultured for an additional 2 h. The cells were subsequently washed twice with PBS and collected by scraping. Approximately 5 × 10^3^ cells in 6 μl of PBS were mixed with 54 μl of Comet Assay LMAgarose (R&D Systems), placed on GelBond Film (Lonza), covered with a 18 mm cover slide (Matsuami), and left at 4 °C for 1 h. Upon removal of the cover slide, the cells were lysed with Comet Assay Lysis Solution (R&D Systems) at 4 °C for 1 h. Following a wash with TBE (90 mM Tris borate [pH 8.3] and 2 mM EDTA), the samples were subjected to electrophoresis at 35 V, for 14 min in TBE. After washing with TBE, samples were fixed with 70% ethanol for 5 min at room temperature and dried overnight. The nuclei were stained with SYBR Green I (Invitrogen) in 10 mM Tris–HCl (pH 7.5) and 1 mM EDTA for 5 min at 4 °C. Images were taken with a fluorescent microscope BZ-X800 (Keyence). Tail moments were measured by using CometScore Pro software (TriTek). At least 140 cells were analyzed per condition from at least three biological repeats.

### Immunofluorescence staining

For investigating BRCA1 foci formation, cells were treated with 20 μM etoposide for 1 h, and then etoposide was washed away with PBS, followed by 6 h incubation. Cells were pre-extracted with 0.2% Triton X-100 for 5 min and then fixed with 3% paraformaldehyde and 2% sucrose in PBS for 15 min. Hereafter, the samples were washed twice with 0.1% Tween-20 in PBS after each procedure. After incubating cells with 2% BSA/PBS for 1 h, the cells were sequentially incubated with primary antibodies overnight and with secondary antibodies for 30 min diluted in 2% BSA/PBS. The samples were sealed with VECTASHIELD (Vector Laboratories), and images were taken with a confocal microscope (FV3000).

### Statistical analysis

Statistical analyses performed were described in each figure legend.

## Data availability

All the data generated or analyzed during the study are included in the article.

## Supporting information

This article contains supporting information.

## Conflict of interest

The authors declare that they have no conflicts of interest with the contents of this article.
