# Supplementary material for: DHX9 phosphorylation at S321 by ATM regulates DHX9 retention at DNA double-strand break sites and interaction with BRCA1
Source: J Biol Chem. 2025 Jul 25;301(9):110526. doi: 10.1016/j.jbc.2025.110526 (PMC12446777; doi:10.1016/j.jbc.2025.110526)
Supplement: Supplementary Table 1 [file mmc1.pdf]

Supplementary Table S1 Antibodies used in this research

Antibody name	Supplier	Catalong number	Dilution ratio	Application
ATM antibody (G-12)	Sant Cruz Biotechnology	sc-377293	200	Immunoblotting
CyclinA antibody (H-432)	Sant Cruz Biotechnology	sc-751	1,000	Immunoblotting
Anti RNA helicaseA antibody	Abcam	ab54593	1,000	Immunoblotting, Immunoprecipitation
Anti-GFP	Roche	11814460001	1,000	Immunoblotting
GFP antibody (B-2)	Sant Cruz Biotechnology	sc-9996	500	Immunoblotting
Anti-Histone H2A.X antibody	Abcam	ab11175	10,000	Immunoblotting
Ku (p80) Ab-2 (Clone 111)	Thermo Fisher Scientific	MS-285-P0	2,000	Immunoblotting
Phospho-Chk1 (Ser345) (133D3) Rabbit mAb	Cell Signaling Technology	2348	1,000	Immunoblotting
Anti-RPA32/RPA2 (phospho S4 + S8) antibody	Abcam	ab87277	1,000	Immunoblotting
Monoclonal Anti-α-Tubulin antibody produed in mouse	Sigma-Aldrich	T9026	5,000	Immunoblotting
Phospho-Histone H2A.X (Ser139) Antibody	Cell Signaling Technology	2577	1,000	Immunoblotting
Phospho-(Ser/Thr) ATM/ATR Substrate Antibody	Cell Signaling Technology	2851	1,000	Immunoblotting
Phospho-KAP-1 (Ser824) Polyclonal Antibody	Thermo Fisher Scientific	IHC-00073	1,000	Immunoblotting
Anti-rabbit IgG, HRP-linked Antibody	Cell Signaling Technology	7074	10,000	Immunoblotting
Anti-mouse IgG, HRP-linked Antibody	Cell Signaling Technology	7076	10,000	Immunoblotting
Anti-Rabbit IgG (Goat), HRP-conjugated, Pre-absorbed	Nacalai tesque	21858-24	10,000	Immunoblotting
DNA-PKcs Ab-1 (Clone 18-2)	Thermo Fisher Scientific	MS-369-P0	1,000	Immunoblotting
BRCA1 Antibody (D-9)	Sant Cruz Biotechnology	sc-6954	2,000	Immunofluorescent staining
CENP-F (D6X4L) Rabbit mAb	Cell Signaling Technology	58982T	250	Immunofluorescent staining
Donkey Anti-Mouse IgG H&L (Alexa Fluor 405)	Abcam	ab175658	1,000	Immunofluorescent staining
Anti-rabbit IgG (H+L), F(ab')2 Fragment (Alexa Fluor 555 Conjugate)	Cell Signaling Technology	4413S	1,000	Immunofluorescent staining
