# Supplementary material for: DHX9 phosphorylation at S321 by ATM regulates DHX9 retention at DNA double-strand break sites and interaction with BRCA1
Source: J Biol Chem. 2025 Jul 25;301(9):110526. doi: 10.1016/j.jbc.2025.110526 (PMC12446777; doi:10.1016/j.jbc.2025.110526)
Supplement: Supplementary Figure 1 [file mmc2.pdf]

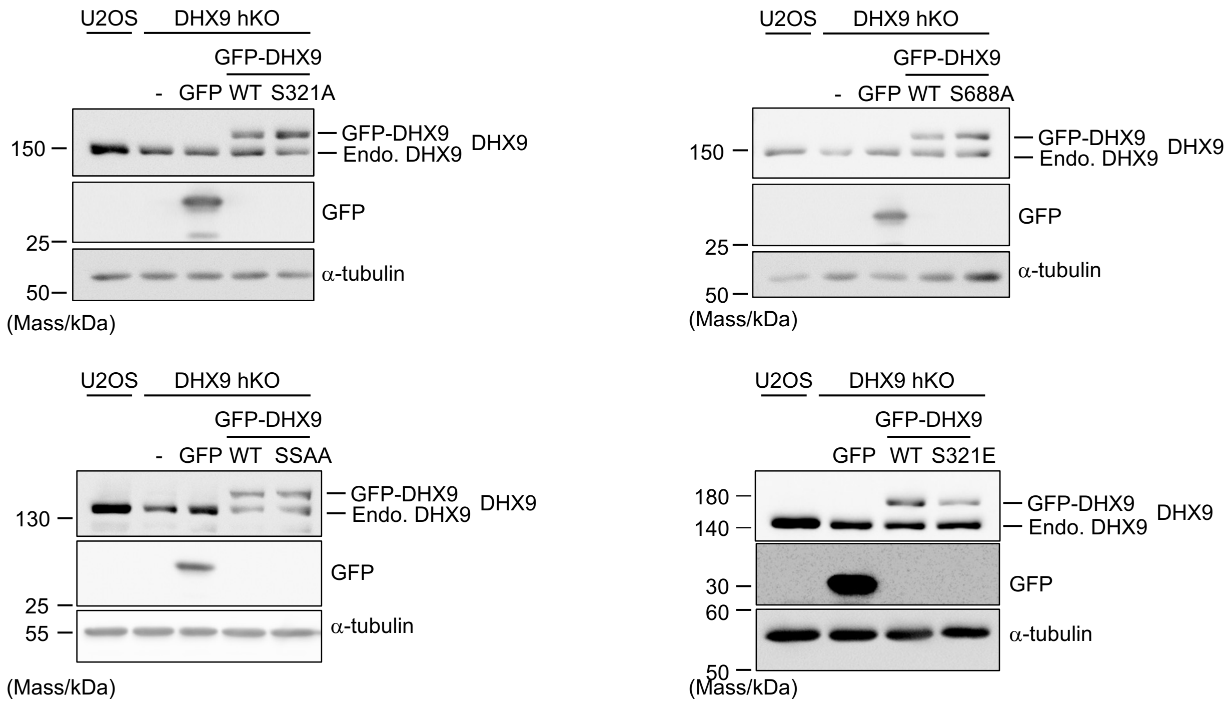

Supplementary Figure S1 Establishment of DHX9 hKO cell lines stably expressing either GFP or GFP-DHX9

Cell extracts of U2OS, DHX9 hKO cells complemented with either GFP or GFP-DHX9 [WT, S321A, S688A, S321AS688A (SSAA) and S321E] were examined by immunoblotting with the indicated antibodies.
