# Supplementary material for: DHX9 phosphorylation at S321 by ATM regulates DHX9 retention at DNA double-strand break sites and interaction with BRCA1
Source: J Biol Chem. 2025 Jul 25;301(9):110526. doi: 10.1016/j.jbc.2025.110526 (PMC12446777; doi:10.1016/j.jbc.2025.110526)
Supplement: Supplementary Figure 2 [file mmc3.pdf]

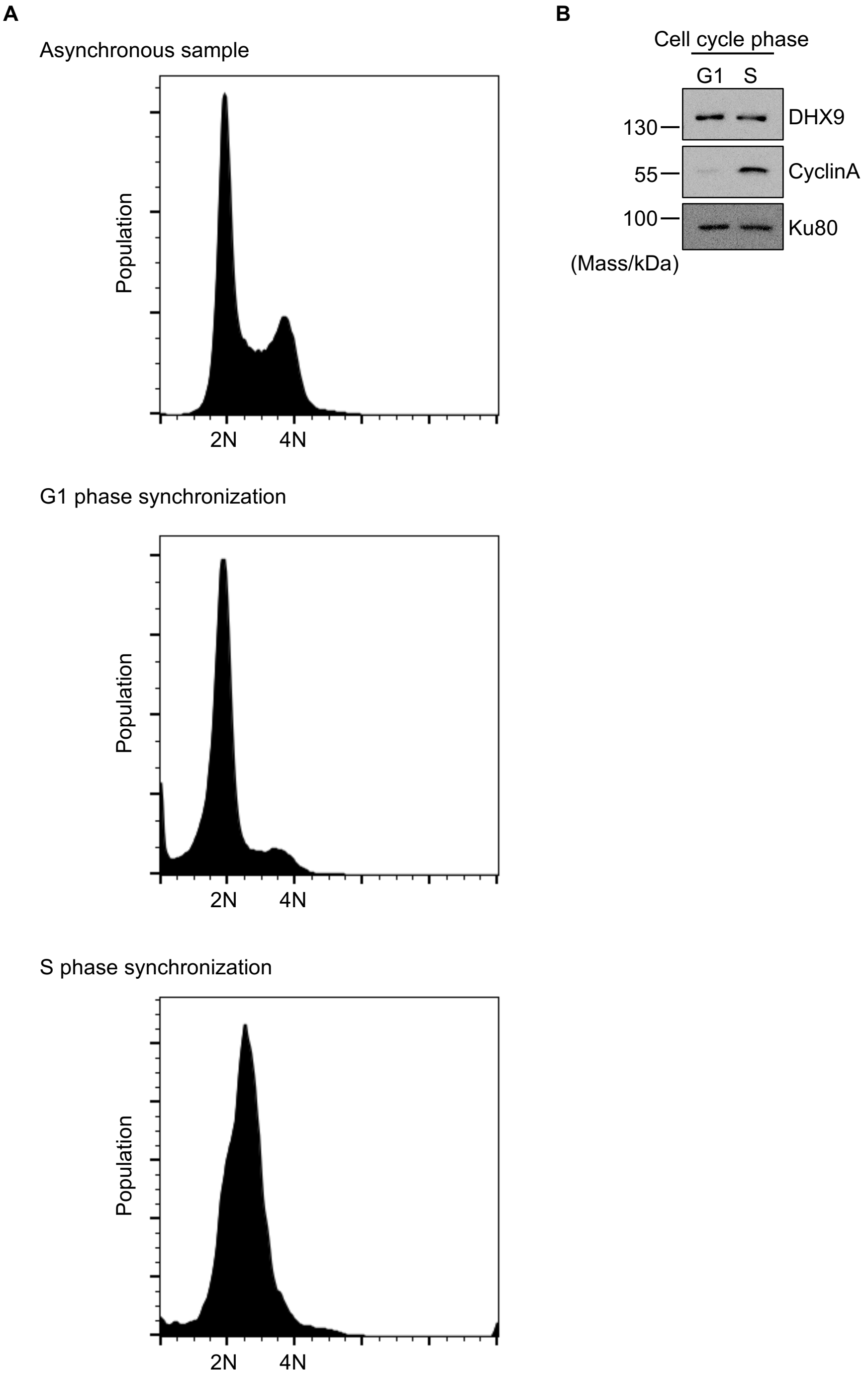


**Supplementary Figure S2 Cell cycle synchronisation analysis**

**(A)** U2OS cells were synchronised at the G1 or S phase by double-thymidine block and following culture as described in materials and methods section. DNA contents and population of the cells were represented as histograms. Asynchronous sample was used as a control.

(B) Whole cell extracts were prepared from U2OS cells synchronised at the G1 or S phase. Expression of endogenous DHX9 was examined by immunoblotting with anti DHX9 antibody. The detection of cyclin A and Ku80 were controls for cell cycle phase and protein loading, respectively.
