# Supplementary material for: DHX9 phosphorylation at S321 by ATM regulates DHX9 retention at DNA double-strand break sites and interaction with BRCA1
Source: J Biol Chem. 2025 Jul 25;301(9):110526. doi: 10.1016/j.jbc.2025.110526 (PMC12446777; doi:10.1016/j.jbc.2025.110526)
Supplement: Supplementary Figure 3 [file mmc4.pdf]

H. sapiens	292	ELNLEILPPP------EDPSVPVALNIGKLAQF-EPSQRQNQVGVVPWSPPQSNWNPWTS	344
M. musculus	295	ELDLEIVPPP------VDPSMPVILNIGKLAHF-EPSQRQNAVGVVPWSPPQSNWNPWTS	347
D. rerio	90	ELGIHIPPPP------QDPNSPVSLVQGKLAHF-EPSQRQSMAGVVPWSPPQVNWNPWTS	142
X. laevis	295	ELGIELPYPP------EDPSQPVSLNLGKLVHF-EPSQKQSHSGVVPWSPPQENWNPWTS	347
D. melanogaster	281	GLDLPVVNPRNIKIELDGPPIPLIVNLSRIDSSQQDGEKRQESSVIPWAPPQANWNTWHA	340

Supplementary Figure S3 Conservation of DHX9 phosphorylation site

Alignment of amino acid sequence around S321 of human DHX9. DHX9 of *H. sapiens*, *M. musculus*, *D. reio*, *X. laevis* and *D. melanogaster* were analysed. Residual numbers of amino acid were shown next to amino acid sequences. S321 and following glutamine (Q) were indicated with the red box.
