# Supplementary material for: DHX9 phosphorylation at S321 by ATM regulates DHX9 retention at DNA double-strand break sites and interaction with BRCA1
Source: J Biol Chem. 2025 Jul 25;301(9):110526. doi: 10.1016/j.jbc.2025.110526 (PMC12446777; doi:10.1016/j.jbc.2025.110526)
Supplement: Supplementary Figure 4 [file mmc5.pdf]

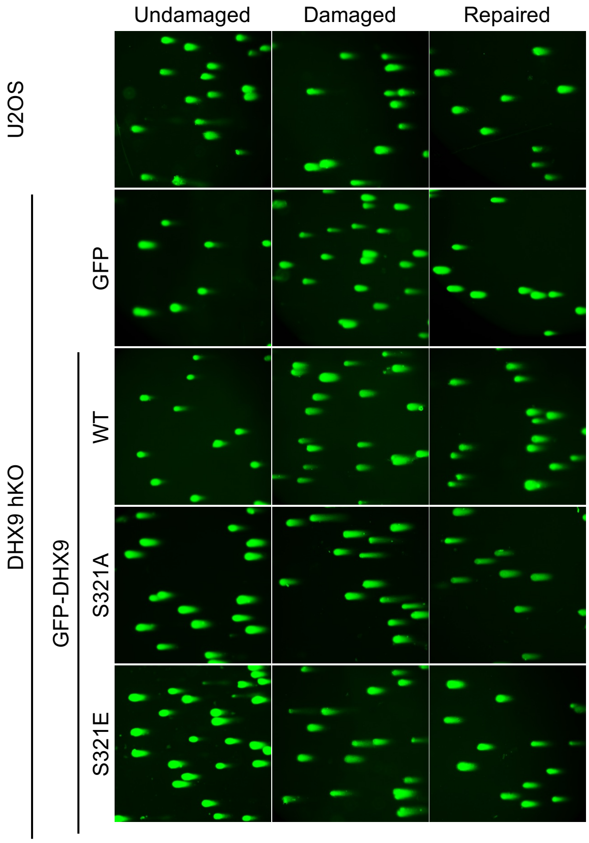

Supplementary Figure S4 Representative images of neutral comet assay

The indicated cells were subjected to neutral comet assay. The representative images of undamaged, damaged and repaired samples were shown.
