# Supplementary material for: DHX9 phosphorylation at S321 by ATM regulates DHX9 retention at DNA double-strand break sites and interaction with BRCA1
Source: J Biol Chem. 2025 Jul 25;301(9):110526. doi: 10.1016/j.jbc.2025.110526 (PMC12446777; doi:10.1016/j.jbc.2025.110526)
Supplement: Supplementary Figure 5 [file mmc6.pdf]

Supplementary Figure S5

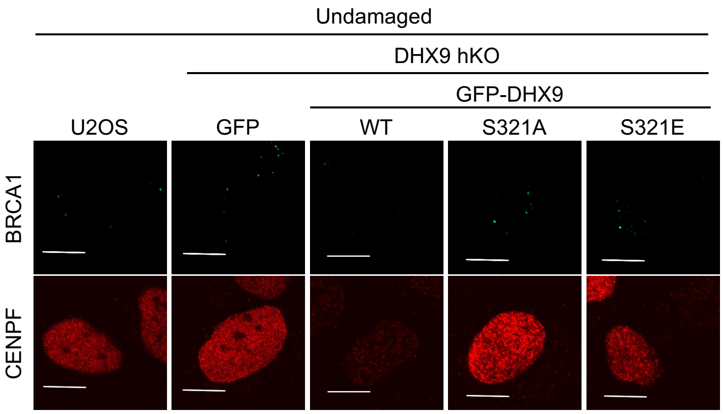

Supplementary Figure S5 Representative images of immunofluorescent staining of mock treated samples

The indicated cells that were mock treated were processed for immunofluorescent staining with the indicated antibodies.
