# Supplementary material for: DHX9 phosphorylation at S321 by ATM regulates DHX9 retention at DNA double-strand break sites and interaction with BRCA1
Source: J Biol Chem. 2025 Jul 25;301(9):110526. doi: 10.1016/j.jbc.2025.110526 (PMC12446777; doi:10.1016/j.jbc.2025.110526)
Supplement: Supplementary Figure 6 [file mmc7.pdf]

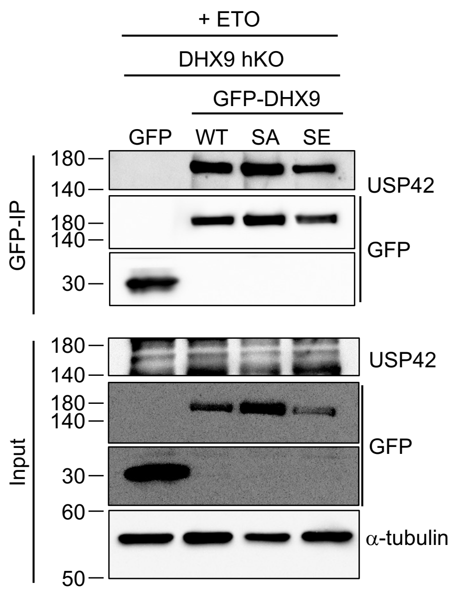

Supplementary Figure S6 Phosphorylation of DHX9 at S321 did not affect the interaction with USP42

The indicated cells were treated with etoposide (50 μM, 1 hour) and then subjected to immunoprecipitation. Input and immunoprecipitated fractions were analysed by immunoblotting with the indicated antibodies.
