# Supplementary material for: DHX9 phosphorylation at S321 by ATM regulates DHX9 retention at DNA double-strand break sites and interaction with BRCA1
Source: J Biol Chem. 2025 Jul 25;301(9):110526. doi: 10.1016/j.jbc.2025.110526 (PMC12446777; doi:10.1016/j.jbc.2025.110526)
Supplement: Supplementary Figure 7 [file mmc8.pdf]

Supplementary Figure S7

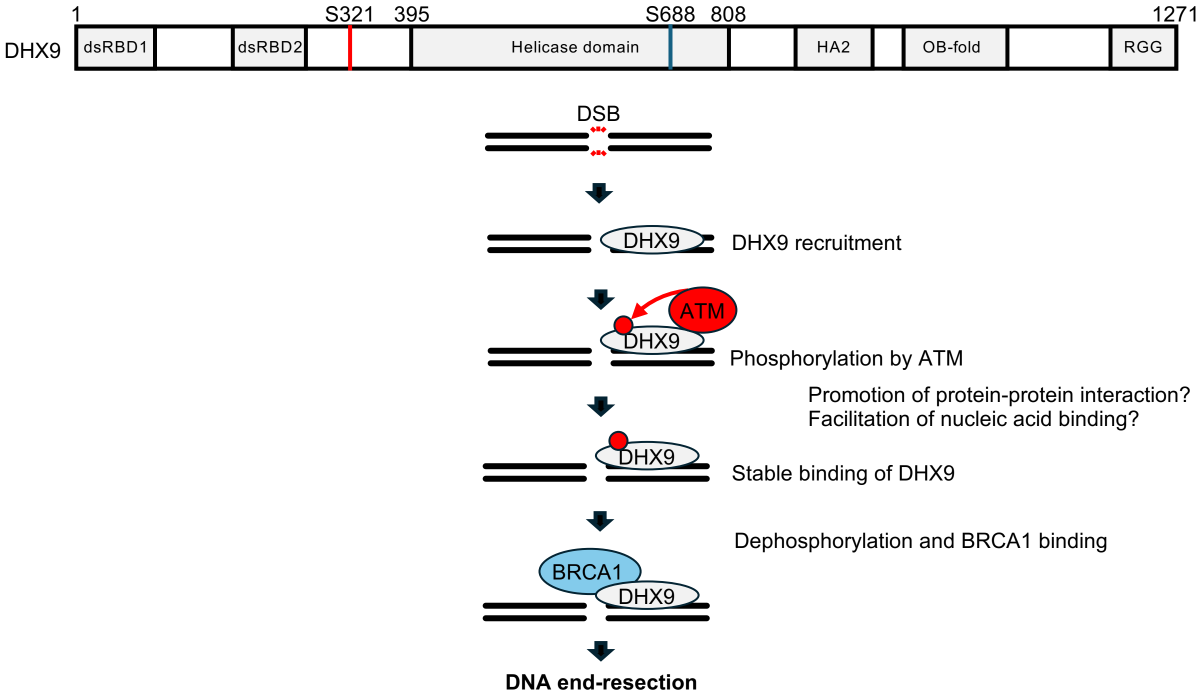

Supplementary Figure S7 A model suggested by the current study
A schematic representation of DHX9 domain structure (upper) and a model indicated by this research (lower). dsRBD: double-strand RNA binding domain, HA2: helicase associated 2, OB-Fold: oligonucleotide/oligosaccharide-binding fold), RGG: arginine-glycine-glycine domain.
